# MicroRNA-218 Is Deleted and Downregulated in Lung Squamous Cell Carcinoma

**DOI:** 10.1371/journal.pone.0012560

**Published:** 2010-09-03

**Authors:** Morgan R. Davidson, Jill E. Larsen, Ian A. Yang, Nicholas K. Hayward, Belinda E. Clarke, Edwina E. Duhig, Linda H. Passmore, Rayleen V. Bowman, Kwun M. Fong

**Affiliations:** 1 Department of Thoracic Medicine, The Prince Charles Hospital, Brisbane, Queensland, Australia; 2 Discipline of Medicine, University of Queensland, Brisbane, Queensland, Australia; 3 Oncogenomics Laboratory, Queensland Institute of Medical Research, Brisbane, Queensland, Australia; 4 Department of Anatomical Pathology, The Prince Charles Hospital, Brisbane, Queensland, Australia; National Cancer Institute, United States of America

## Abstract

MicroRNAs (miRNAs) are a family of small, non-coding RNA species functioning as negative regulators of multiple target genes including tumour suppressor genes and oncogenes. Many miRNA gene loci are located within cancer-associated genomic regions. To identify potential new amplified oncogenic and/or deleted tumour suppressing miRNAs in lung cancer, we inferred miRNA gene dosage from high dimensional arrayCGH data. From miRBase v9.0 (http://microrna.sanger.ac.uk), 474 human miRNA genes were physically mapped to regions of chromosomal loss or gain identified from a high-resolution genome-wide arrayCGH study of 132 primary non-small cell lung cancers (NSCLCs) (a training set of 60 squamous cell carcinomas and 72 adenocarcinomas). MiRNAs were selected as candidates if their immediately flanking probes or host gene were deleted or amplified in at least 25% of primary tumours using both Analysis of Copy Errors algorithm and fold change (≥±1.2) analyses. Using these criteria, 97 miRNAs mapped to regions of aberrant copy number. Analysis of three independent published lung cancer arrayCGH datasets confirmed that 22 of these miRNA loci showed directionally concordant copy number variation. MiR-218, encoded on 4p15.31 and 5q35.1 within two host genes (*SLIT2* and *SLIT3*), in a region of copy number loss, was selected as a priority candidate for follow-up as it is reported as underexpressed in lung cancer. We confirmed decreased expression of mature miR-218 and its host genes by qRT-PCR in 39 NSCLCs relative to normal lung tissue. This downregulation of miR-218 was found to be associated with a history of cigarette smoking, but not human papilloma virus. Thus, we show for the first time that putative lung cancer-associated miRNAs can be identified from genome-wide arrayCGH datasets using a bioinformatics mapping approach, and report that miR-218 is a strong candidate tumour suppressing miRNA potentially involved in lung cancer.

## Introduction

MiRNAs are a highly conserved family of small non-coding RNA species that negatively regulate gene expression via RNA interference. Calin *et al* first identified two miRNAs (miR-15 and miR-16) within 13q14, a region frequently deleted in chronic lymphocytic leukaemia, with significantly reduced expression [1]. Since then, altered miRNA expression has been reported in numerous malignancies, including colon [2], [3], breast [4], [5], lung [6], [7], [8], liver [9], brain [10], [11], lymphoma [12], [13], [14] and leukaemia [1], [15], [16].

In lung cancer, studies have identified the tumour suppressing *let-7* family and the oncogenic *miR-17-92* cluster of miRNAs. The *let-7* family is consistently downregulated in primary lung tumours and lung cancer cell lines, which abrogates *let-7's* control on *RAS*, allowing *RAS* overexpression, thereby contributing to lung carcinogenesis [6], [7], [17]. Significant overexpression of the oncogenic *miR-17-92* cluster of miRNAs occurs in lung cancer cell lines and tumours [18], where *miR-17-92* miRNAs are upregulated by oncogenic c-Myc and act as part of a regulatory network balancing cell death and proliferation with c-Myc and E2F1 [19], [20], [21].

MiRNAs demonstrate complex patterns of genomic organisation with both intergenic and intragenic miRNA genes [22], [23]. Calin *et al* reported that most known miRNAs are in regions of genomic aberration associated with cancer [17]. In keeping, *let-7* and *miR-17-92* are linked to chromosomal deletions and gains respectively, indicating copy number variations as potential mechanisms for their dysregulation in lung tumours [17], [21], [24].

We reasoned that novel dysregulated lung cancer miRNAs can be identified by virtue of somatically acquired aberrant gene dosage, and this allows the large number of publically available unbiased genome-wide arrayCGH datasets to be exploited for identification of miRNAs involved in disease even though the original studies were not specifically designed for miRNA loci. Using this strategy we report for the first time, identification of miR-218 (hsa-miR-218; MIMAT0000275) located within a region of genomic loss (4p15.31 and 5q35.1) as a putative tumour suppressor in non-small cell lung cancer (NSCLC). MiR-218 was recently identified as a tumour suppressor in cervical cancer [25] where its downregulation was linked with human papilloma virus (HPV) [25]. Although we observed a significant reduction in miR-218 in subjects with a history of cigarette smoking we found no relationship between HPV and miR-218 in lung SCCs.

## Materials and Methods

### Clinical Specimens

Resected primary NSCLC and corresponding normal lung tissue were obtained with informed written consent from patients undergoing lung resection at The Prince Charles Hospital between 1990 and 2007 (Human Research Ethics Committee 9124). Clinicopathological data was available for all specimens as previously published including disease recurrence [26], [27] and asbestos fibre burden [28].

### ArrayCGH Detection of Chromosomal Aberration

ArrayCGH data for 132 curatively resected primary NSCLCs (training set) was provided by Dr JE Larsen (manuscript in preparation) (Table 1) using the Agilent Human Genome CGH Microarray 44B (Agilent, G4410B) platform and tumour sample hybridisation against normal female DNA (Human Genomic DNA Female, Promega G152A) according to the manufacturer's instructions. Filtered, normalised signal log ratios between lung tumour DNA and normal female reference DNA were used for analyses.

**Table 1:** Clinicopathological characteristics of arrayCGH cohort.

		Squamous Cell Carcinoma (n = 60)	Adenocarcinoma (n = 72)	All Primary NSCLCs (n = 132)
**Age** (years)		66.2 (38.9–83.7)	64.7 (35.9–81.6)	**65.4 (35.9–83.7)**
**Sex**	Male	43	49	**92**
	Female	17	23	**40**
**Stage**	Stage I	29	53	**82**
	Stage II	23	10	**33**
	Stage III	7	5	**12**
	Stage IV	1	4	**5**
**Smoking Status** a	Current	26	30	**56**
	Former	31	36	**67**
	Never	3	6	**9**

a Former smokers had quit smoking at least one year prior to lung resection, while current smokers continued smoking within one year of lung resection. Pack years were calculated as number of cigarettes per day, multiplied by number of years of smoking, divided by 20. Never smokers had smoked less than 100 cigarettes in their life-time.

For robust detection of DNA copy number aberrations, two independent bioinformatic approaches were used: i) Fold change (FC) of +/−1.2 (high discovery sensitivity) in individual probe intensity using signal log ratios (Affymetrix, Statistical Algorithms Reference Guide); and ii) CGH Explorer Analysis of Copy Errors (ACE) algorithm [29] controlling significance and false discovery rate at <0.001 to avoid Type 1 error for multiple comparisons. Adenocarcinomas (AC) (n = 72) and squamous cell carcinomas (SCC) (n = 60) were analysed separately. Analysis of chromosome X was conducted in the female data set (AC n = 23 and SCCs n = 17). Chromosome Y, which does not contain any known miRNA genes, was not examined. Selected thresholds for all analyses are summarised in Table S1.

### Identifying miRNAs within Regions of ArrayCGH Detected Chromosomal Aberration

474 human miRNAs from Sanger miRBase Version 9.0 (http://microrna.sanger.ac.uk/) [30], [31], [32] were physically mapped onto the arrayCGH dataset using miRNA precursor chromosome number and nucleotide position, effectively positioning them between proximal and distal flanking arrayCGH probes.

MiRNA genes were classified as having copy number aberrations if both flanking arrayCGH probes were: i) located within 35kb (average spatial resolution of the platform) of the miRNA or positioned within the miRNAs host gene; and ii) identified as concordant gain or loss by both bioinformatic methods (i.e. FC and ACE). These were then called as “significant” if both flanking probes were concordantly altered in ≥25% but discordant in <10% of primary tumours. This produced a list of miRNAs in regions of copy number variation.

### Validation of miRNAs from Independent Test Sets

We also interrogated published arrayCGH data from three independent primary NSCLC cohorts [33], [34], [35] (Table S2). Regions of chromosomal aberration were compiled and miRNAs in areas of gain or loss were identified using genomic positioning information as described above. This list of miRNAs was then directly compared with candidates derived from the TPCH (training) dataset to select those miRNAs within regions of aberration consistent in the training set and at least one test set.

### Prioritising miRNAs with Gene Dosage and Expression Concordance

To select candidate miRNAs with concordantly altered dosage and expression, we interrogated four public datasets of mature miRNA expression from independent NSCLC cohorts [6], [7], [8], [36] (Table S3). Candidate miRNAs with changes in expression that were concordant with their inferred copy number changes were prioritised for further study.

### Biological Verification of Aberrant miRNA Expression

Mature miRNA expression was measured using TaqMan qRT-PCR assays (Applied Biosystems) in 39 paired NSCLCs and normal lung (SCCs n = 18 and ACs n = 21) (Table 2). Total RNA was isolated from 30 mg of tissue using TRIzol (Invitrogen) and DNase treated (Ambion). MiRNA-specific reverse transcription for miR-218 and U6 snRNA (internal control) used stem-loop TaqMan primer/probe sets for real-time PCR (qRT-PCR) [37]. Triplicate TaqMan qRT-PCR assays were performed on a Corbett Research Rotor-Gene 6000 with reference total RNA certified to contain miRNAs (FirstChoice Human RNA Survey Panel, Ambion). Reaction efficiency and linear dynamic range was determined for each assay. A ratio of miRNA expression to U6 snRNA expression was obtained using the Pfaffl method [38].

**Table 2:** Clinicopathological characteristics of miR-218 validation cohort.

		Squamous Cell Carcinoma	Adenocarcinoma	All Primary NSCLCs
		(n = 18)	(n = 21)	(n = 39)
**Age** (years)		66.5	67.6	**67.1**
(range)		(44.4–83.7)	(47.3–81.6)	**(44.4–83.7)**
**Sex**	Male	12	14	**26**
	Female	6	7	**13**
**Stage**	Stage I	5	15	**20**
	Stage II	11	2	**13**
	Stage III	2	3	**5**
	Stage IV	0	1	**1**
**Smoking**	Current	7	7	**14**
**Status** a	Former	8	11	**19**
	Never	3	3	**6**
**Asbestos Exposure**	Exposed (>20AB/gww)	5	10	**15**
	Low Exposure (1–20AB/gww)	0	1	**1**
	No Exposure (0AB/gww)	13	10	**23**
**HPV DNA**	HPV 16	0	–	**–**
	HPV 18	1	–	**–**
	HPV 31	1	–	**–**
	Negative	16	–	**–**
**Recurrence**	Rec (3–18 months)	7	12	**19**
	Non-Rec (>36 months)	8	7	**15**
	Excluded	3	2	**5**
**Survival**	Alive	7	6	**13**
	Deceased	11	15	**26**
	Survival Duration (months) (range)	24.7 (6.6–62.4)	25.3 (4.3–84.6)	**25.1 (4.3–84.6)**

a Former smokers had quit smoking at least one year prior to lung resection, while current smokers continued smoking within one year of lung resection. Pack years were calculated as number of cigarettes per day, multiplied by number of years of smoking, divided by 20. Never smokers had smoked less than 100 cigarettes in their life-time. Abbreviations: Rec, Recurrence; Non-Rec, Non-Recurrent.

### Correlating Dysregulated miRNA and Host Gene Expression

Host gene expression was measured by qRT-PCR using SYBR Green chemistry in the same cohort of 39 NSCLCs. DNase treated total RNA were reverse transcribed using Superscript III (Invitrogen). Primers were designed using Primer Express (Applied Biosystems) and are listed in Table S4. Standard curves were performed to determine the linear dynamic range and reaction efficiency. All reactions were performed in triplicate on a Corbett Rotorgene 6000 with melt curve analysis to confirm detection of a single amplicon, with Universal Human Reference RNA (Stratagene) as reference. Relative gene expression was calculated and normalised against internal controls (18S rRNA, ACTN4 and BAT1) [39], using the Pfaffl method [38].

### HPV Testing

Presence of HPV DNA was assessed in a cohort of 72 lung SCCs accessed from TPCH Tumour Bank, including the 18 cases with miR-218 expression data. HPV testing was performed by Gribbles Pathology, Victoria, Australia, using the Genera Biosystems PapType RUO Kit which detects 14 high-risk (16, 18, 31, 33, 35, 39, 45, 51, 52, 56, 58, 59, 66 and 68) and two low risk (6 and 11) HPV subtypes. This assay is based on 2 rounds of PCR amplification of HPV and control gene DNA (alkali myosin light chain protein) with addition of a fluorescent reporter dye and utilises silica detection beads which fluoresce when hybridised to its specific target DNA.

### Statistical Analysis

Statistical analyses were performed as above or with SPSS Version 13.0 (SPSS Inc Chicago, IL, USA) using Mann-Whitney *U* and Pearson correlations. p values <0.05 were considered statistically significant. Survival analysis was undertaken with log rank statistic of constructed Kaplan-Meier curves.

### MiRNA Target Prediction and Pathway Analysis

Predicted mRNA targets of miR-218 were identified by using four online miRNA target prediction programs: PicTar (5 species conservation) [40], TargetScan 4.1 [41], , miRBase Targets Version 5 [30], [31], [32] and miRNAMap 2.0 [44], [45]. Over-represented gene ontologies (GO) and functional classes for putative target genes of miR-218 predicted by two or more of the algorithms were identified using the Database for Annotation, Visualization and Integrated Discovery (DAVID, April 2008 release) gene-GO enrichment analysis (ranked by the Expression Analysis Systematic Explorer (EASE) score threshold) and gene functional classification (performed with the highest stringency setting) [46], [47]. Ingenuity Pathway Analysis (Ingenuity® Systems, www.ingenuity.com) was performed on both the entire miR-218 target gene list and a prioritised list of miR-218 targets found to be enriched ≥2.0 by DAVID gene functional classification.

## Results

### MiRNAs are Located in Regions of Genomic Alteration in Lung Cancer

From TPCH arrayCGH data (training set), 89 of 474 (19%) miRNAs were within chromosomal regions deleted (41 miRNAs) or gained (48 miRNAs) in 132 primary NSCLC (Table S5). Within the SCC subtype, 77 miRNAs were associated with DNA copy number changes, with 40 miRNAs in areas of gain and 37 in regions of loss. Twenty-six miRNAs were identified for ACs, with 21 and five miRNAs in regions of gain and loss respectively. Fourteen miRNAs were located within regions of copy number variation common to both SCC and ACs (one associated with DNA loss and 13 associated with DNA gain). On the X chromosome, five and four miRNAs were found to be associated with regions of loss in ACs and SCCs respectively.

### Validation of Candidate NSCLC miRNAs from Public Data Test Sets

#### MiRNA dosage

**Figure 1:**
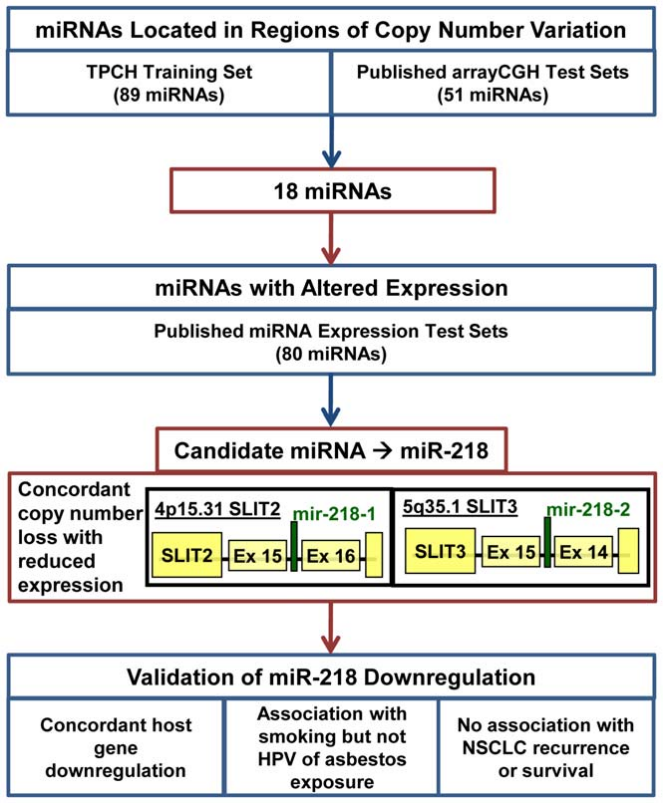
Identification of miR-218 from concordant copy number variation and gene expression from independent data sets. Expression levels of 38/89 candidate miRNAs and 19/51 published arrayCGH miRNAs have not been studied due to their recent inclusion in miRBase. The genomic location of miR-218 within its two host genes, SLIT2 and SLIT3, is also illustrated.

To validate candidate miRNAs, we repeated the positional mapping analysis using published independent arrayCGH data test sets [33], [34], [35] and found 51 miRNAs; 28 and 23 miRNAs located within regions of gain or loss respectively (Table S6). Eighteen of these were among the 89 miRNAs identified from positional mapping to arrayCGH (Figure 1). No miRNAs were found to be located in genomic regions of copy number variation that were discordant between our cohort (training set) and published arrayCGH cohorts (test sets).

#### MiRNA Expression

To enrich for miRNAs with concordant dysregulated expression and dosage, we reviewed the literature and found 80 mature miRNAs reported to have altered expression levels (44 increased, 32 decreased and four with conflicting reports) in primary NSCLCs [6], [7], [8], [36], 16 were represented in the 89 miRNA set with 10 showing expression dysregulation concordant with predicted miRNA gene copy number change. Seven miRNAs were among the 51 miRNAs identified from published arrayCGH studies, but only three had concordant miRNA expression and dosage between the public datasets. Two miRNAs, miR-218 and miR-216, were common to training and all test sets, however, only miR-218 demonstrated concordant loss of copy number and expression (Figure 1). Expression levels of 38/89 candidate NSCLC miRNAs and 19/51 published arrayCGH NSCLC miRNAs have only recently been annotated in miRBase and have not yet been studied in lung cancer.

### MiR-218 Host Genes are in Regions of Copy Number Loss in NSCLC

MiR-218 is produced from two unique miRNA precursors (hsa-mir-218-1; MI0000294 and hsa-mir-218-2; MI0000295), encoded in separate genomic locations, 4p15.31 and 5q35.1 respectively. Both precursors are intragenic, with mir-218-1 situated within intron 15–16 or 14–15 of *SLIT2* (Slit Homolog 2) and mir-218-2 residing within intron 14–15 of *SLIT3* (Slit Homolog 3) (Figure 1). Mir-218-2 was identified as within a region of copy number loss in >25% of SCCs but only >15% of ACs with average fold losses of *SLIT3* flanking probes −1.205 (range −2.23 to −1.11) and −1.115 (range −1.85 to +1.39) respectively. However, mir-218-1 was located in a region of loss unique to SCCs, with an average fold loss of *SLIT2* flanking probes −1.13 (range −1.95 to +1.29) (Table S7).

### MiR-218 Expression is Reduced in NSCLCs Compared with Paired Normal Lung

**Figure 2:**
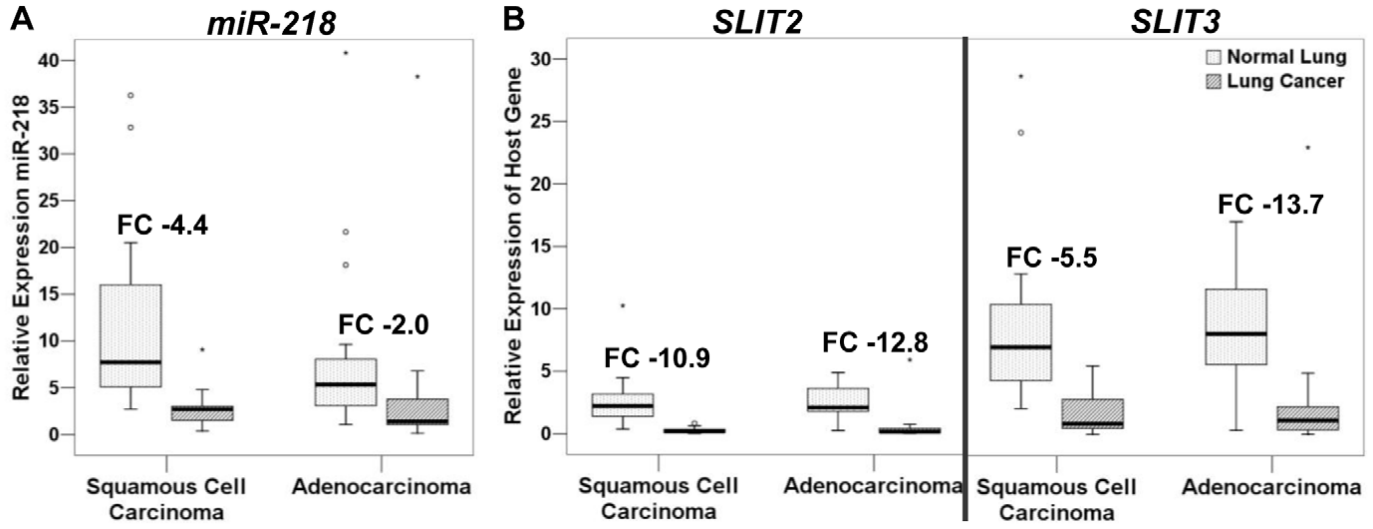
Expression of miR-218 and its host genes in primary NSCLCs and their paired normal lung. *p<0.05; **p<0.01. (a) Down-regulation of miR-218 expression was observed in both ACs (FC -2; p = 0.001; Z score  = −3.258) and SCCs (FC −4.365; p<0.0001; Z score  = −4.406). (b) A reduction in host gene expression was observed in both ACs (*SLIT2*: FC −12.77, p<0.0001, Z score  = −4.277; *SLIT3*: FC −13.73, p<0.0001, Z score  = −3.839) and SCCs (*SLIT2*: FC −10.85, p<0.0001, Z score  = −5.182; *SLIT3*: FC −5.46, p<0.0001, Z score  = −4.540). Abbreviations: FC, Fold change.

To confirm decreased miR-218 dosage and expression in lung cancer, we measured mature miR-218 expression in 21 ACs and 18 SCCs from our arrayCGH training set and their paired normal lung. MiR-218 expression was down-regulated in 85% (33/39) of NSCLC tumours compared with paired normal lung. Statistically significant decreases were observed in both SCCs (mean FC = −4.4, p<1.0e-4) and to a lesser extent ACs (mean FC = −2.0, p = 0.001) (Figure 2a).

### Reduced miR-218 Expression is Associated With *SLIT2* and *SLIT3* Copy Number Loss

Correlation of miR-218 expression with host gene (*SLIT2* and *SLIT3*) arrayCGH probe copy number found the majority of samples (31/39, 79.5%) demonstrated miR-218 down-regulation with a decrease in *SLIT2* and/or *SLIT3* copy number however the correlation was not significant (p>0.05; Pearson) (Figure S1a). Complete concordance, that is, a reported loss of both host genes plus a decrease in miR-218 expression, was observed in 55.6% (10/18) SCCs and 38.1% (8/21) of ACs.

### Concordant Downregulation of miR-218, *SLIT2* and *SLIT3* mRNA Expression

To confirm predicted host gene down-regulation, we measured *SLIT2* and *SLIT3* expression in 37 of the 39 NSCLC samples and compared it with their paired normal lung tissue (19 ACs and 18 SCCs). As expected, *SLIT2* and *SLIT3* down-regulation was observed in 36/37 and 34/37 NSCLCs respectively, with statistically significant down-regulation of both *SLIT2* and *SLIT3* expression in both ACs and SCCs (p<0.001) (Figure 2b).

*SLIT2* copy number loss plus reduced expression was observed in 83% (15/18) SCCs and 79% (15/19) AC. Whilst *SLIT3* demonstrated copy number and expression concordance in 83% (15/18) SCCs but only 53% (10/19) ACs. For both *SLIT2* and *SLIT3*, the relationship between host gene expression and copy number was not significant (p>0.05; Pearson) (Figure S1b).

To determine whether this intragenic miRNA is co-regulated with host gene expression, we examined miR-218 and *SLIT2*/*SLIT3* expression levels. Concordant down-regulation of miR-218, *SLIT2* and *SLIT3* was observed in 15/18 (83.3%) SCCs and 15/19 (79%) ACs. Notably, one AC had concordant increases in miR-218, *SLIT2* and *SLIT3* expression with CN loss of only *SLIT3*. In SCCs, miR-218 was moderately correlated with *SLIT2* (R = 0.496, p = 0.036) and *SLIT3* (R = 0.459, p = 0.055), however, only *SLIT2* was statistically significant. For ACs, weak-moderate correlations were detected (*SLIT2*: R = 0.378, p = 0.111; *SLIT3*: R = 0.297, p = 0.216) but these were not statistically significant (Figure S1c).

### MiR-218, *SLIT2* and *SLIT3* Downregulation is Associated with *SLIT2* and *SLIT3* Gene Dosage

Next, we compared *SLIT2* and *SLIT3* copy number with miR-218, *SLIT2* and *SLIT3* expression to determine if their expression was due to gene dosage. The 15 ACs and 15 SCCs found to have concomitant reduced miR-218 and host gene expression also demonstrated copy number loss in *SLIT2* and/or *SLIT3*. Complete concordance (loss of both host genes plus a decrease in miR-218 expression) between *SLIT2* and *SLIT3* copy number and miR-218, *SLIT2* and *SLIT3* expression, was observed in 9/19 (47.4%) ACs and 10/18 (55.6%) SCCs (Figure S2 and Table S8). For one AC and two SCCs, miR-218 expression was increased despite reduced host gene copy numbers and expression.

### Association of miR-218 with Clinicopathological Phenotypes

#### Smoking History

**Figure 3:**
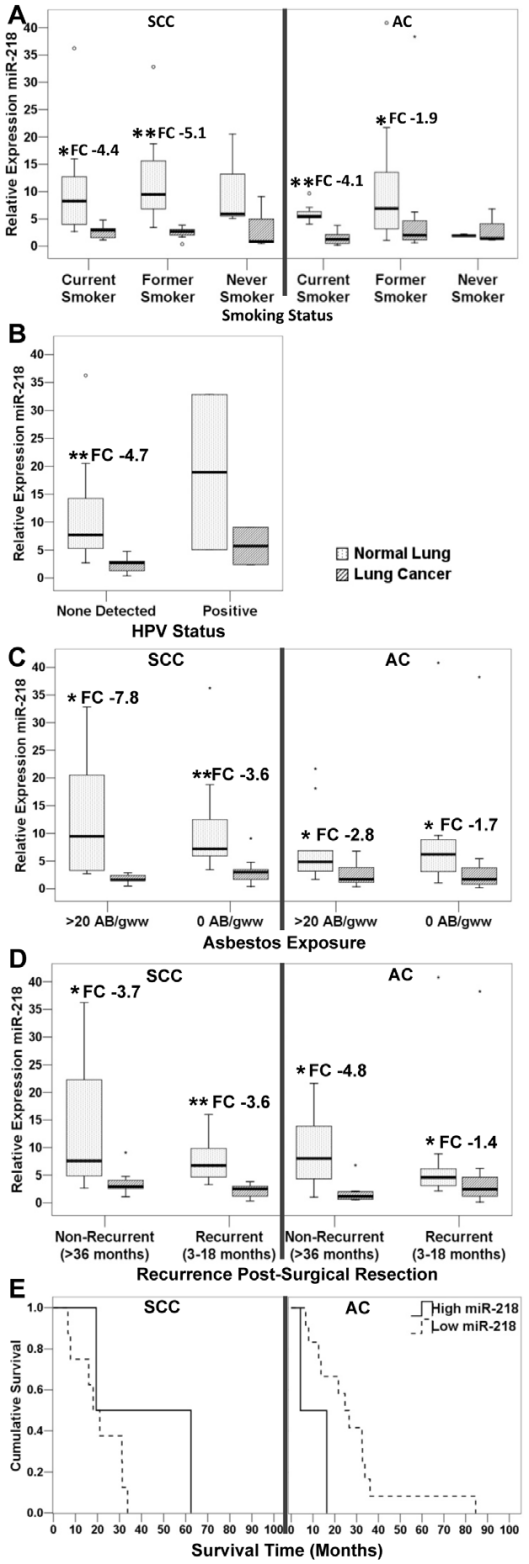
Relationship of miR-218 expression to smoking (a), HPV (b), asbestos (c), recurrence (d) and survival (e). *p = 0.05; **p = 0.01. Abbreviations: FC, Fold change. Statistically significant downregulation of miR-218 expression was observed in current and former smokers of both NSCLC subtypes. No significant associations were observed between miR-218 expression and HPV, asbestos, disease recurrence or survival.

Compared to normal lung tissue, miR-218 expression was significantly decreased in tumours of current (SCC: FC-4.4; AC: FC-4.1; p<0.05) and former (SCC: FC-5.1; AC: FC-1.9; p<0.05) smokers for both subtypes of NSCLC (Figure 3a) but no decrease was observed in never smokers (albeit small sample: SCC n = 3, ACs n = 3). No significant associations were demonstrated between miR-218 expression and total number of pack years smoked (in current and former smokers) or time since quitting smoking (former smokers) in SCCs or ACs (data not shown).

#### HPV Status

A large cohort of SCCs (n = 72) were screened to detect the presence of HPV DNA, with 9% (8/72) of these lung SCCs testing positive. HPV subtypes identified were potentially oncogenic, including types 16 (4/8), 18 (3/8) and 31 (1/8). For 18 SCCs with miR-218 expression data, 9% (2/18) tested positive for HPV (subtypes 18 and 31) (Table 2) and HPV status not found to be associated with a reduction in miR-218 expression (Figure 3b).

#### Asbestos

Altered miR-218 expression in NSCLC was also examined in relation to asbestos exposure. Asbestos fibre burden has previously been assessed for this cohort, and tumours with >20 asbestos bodies per gram wet weight lung tissue (AB/gww) were considered ‘asbestos exposed’ and those with 0 AB/gww were considered to be ‘not exposed’ [28]. Altered miR-218 expression was not found to be associated with asbestos exposure (Figure 3c) nor asbestos fibre burden (number of AB/gww lung tissue - data not shown).

#### Recurrence and Survival

Following the earlier publication by Larsen *et al* [26], [27], recurrent NSCLCs were those that had recurred within 3–18 months post-surgical resection and non-recurrent subjects were those who had remained disease free for a period of at least 36 months post-resection. No association was observed between miR-218 expression levels and disease recurrence or NSCLC survival post-resection (Figure 3d–e).

### Putative Targets of miR-218 Support its Role in Carcinogenesis

We identified predicted target genes of miR-218 using four online miRNA target prediction algorithms. PicTar [40] and miRNAMap 2.0 [44], [45] identified 575 and 688 target genes respectively, with 645 conserved sites and 133 poorly conserved sites. TargetScan 4.1 [41], [42], [43] and miRBase Targets V5 [30], [31], [32] reported 570 and 946 miR-218 target sites respectively, where multiple target sites could be present in the one gene. In total, 1794 individual target genes were identified by these four target prediction algorithms (578 identified by two or more of the algorithms) (available on request).

Gene ontologies and biological function of the 578 target genes were explored using DAVID (April 2008 Release) to determine biological relevance [46], [47]. GO enrichment analysis revealed that biological processes such as cell adhesion, protein modifications and transport, development and cell signalling were over-represented amongst miR-218 targets (p<0.001). Over-represented molecular functions (p<0.01) were predominantly focused on protein binding function, in particular to actin, cytoskeletal proteins, cyclic nucleotides, protein phosphatase 2A, cAMP, and metal ions. Similar to gene ontology analysis, functional annotation clustering reflected enrichment of groups of genes largely involved in cell adhesion (enrichment score  = 4.66), protein modification relating to ubiquitin cycle (enrichment scores  = 3.67 and 3.33) or kinase activity (enrichment score  = 2.88), and regulation of transcription (including proto-oncogenes, enrichment score  = 2.22) (Table S9).

Network analysis of the 578 target genes by Ingenuity Pathway Analysis identified 39 gene networks involved in biological functions such as cell-to-cell signalling, amino acid metabolism, post-translational modifications, cancer and respiratory system development and function (p<0.05) (Figure S3a and Table S10). Canonical pathway analysis found numerous cancer signalling pathways including Wnt/β-catenin signalling, ERK/MAPK signalling and Notch signalling (p<0.05) (Figure S3b). To attempt to define a more specific network or pathway associated with miR-218, we performed a focused analysis using 121 enriched genes (≥2.00) identified with functional annotation clustering in DAVID. This identified 10 gene networks that reflected similar cancer-related biological functions to those identified using the larger target gene list (Table S10). For instance, in the ‘gene expression, cancer and cell morphology’ network, miR-218 may target genes directly and indirectly linked to two oncogenes, *MYC* and *SRC* (Figure S4).

## Discussion

We demonstrate that genomic profiling can be used to identify cancer related miRNAs. We found 19% of miRNAs were located in regions of copy number variation in primary NSCLC, which is relatively lower than ovarian (37%), breast (73%) and melanoma (86%) [48]. Aside from being different tumour types, this may be due to more recently identified miRNAs not highly represented in cancer-associated genomic regions, our high stringency bioinformatics, or both. We limited ourselves to miRNAs located within host genes or next to flanking probes that were within the spatial resolution of the platform (35 kb), because the array used in this study did not have any probes directly representing any miRNA genes. Next generation higher-resolution arrayCGH platforms will refine this approach specifically detecting miRNA genomic loci. Nonetheless, we successfully identified 89 potential lung cancer miRNAs, including established oncogenic and tumour suppressing miRNAs. Three members of the known tumour suppressing *let-7* family of miRNAs were identified in regions of loss (let-7g, let-7f-2 and miR-98), and oncogenic miR-21 was found within a genomic region of amplification.

Over half of the 89 miRNAs (65%) are intragenic; interestingly, many of the host genes have reported roles in cancer. For example, *MCM7* (minichromosome maintenance protein 7) is the host gene for three of the miRNAs (miR-25, miR-93 and miR-106b) we identified and has been reported to be amplified or over-expressed in human malignancies including prostatic, pancreatic, thyroid, cervical and colorectal cancers [49], [50], [51], [52], [53], [54]. In prostate cancer, increased levels of these 3 miRNAs occurred with amplified and over-expressed MCM7 [55]. This observation supports the idea proposed that miRNAs and their host genes may be jointly affected by copy number changes [48].

Only one of the 89 miRNAs, miR-218, demonstrated concordant changes in copy number and expression. Additional candidates may be identified with publication of new miRNA expression studies in NSCLC as half of the miRNAs with concordant copy number changes (10/18) were excluded from this first pass analysis as their mature expression levels have not been measured. Furthermore, miRNAs are subject to complex regulatory mechanisms so it is not surprising to observe numerous miRNAs with discordant copy number and expression (such as miR-216). However, as with any genome-wide approach for gene discovery, there is the potential to identify false positives. We have attempted to alleviate this risk by using high stringency criteria for identification of candidate miRNAs from our aCGH data and subsequently using published NSCLC cohorts with copy number and miRNA expression data to independently validate these candidates. As such, miR-218 emerged as a strong candidate tumour suppressor, within a region of copy number loss in greater than two NSCLC studies and with demonstrated loss of expression in a third independent cohort.

Produced from two separate precursors, miR-218 is found within host genes *SLIT2* and *SLIT3*, with both the miRNA and its host genes demonstrating reduced expression in the majority (80%) of NSCLCs. Zhang *et al* reported copy number losses of mir-218-1 and *SLIT2* in ovarian (16%), breast (36%) and melanoma (33%), however, the alternative precursor and host gene were not mentioned [48]. It has been suggested that intronic miRNAs share host gene transcriptional regulatory control with resulting co-expression [22], [23]. Despite noting a reduction in both miR-218 and host gene expression in the majority of NSCLCs, only a moderate correlation between miR-218 and *SLIT2* expression was detected, with no significant relationship with *SLIT3*. This may be a reflection of disproportionate production of miR-218 from each genomic site, alterations in miRNA biogenesis or miR-218 may have a promoter separate from its host gene. Similarly, no significant associations between miR-218 or host gene expression with host gene copy number were identified, but we found that complete concordance (copy number loss of both host genes with reduced miRNA and host gene expression) observed in 51% of lung cancers studied. These findings would suggests that genetic loss may contribute to the observed reduction in miR-218, *SLIT2* and *SLIT3* expression, however it is likely that aberrations in multiple regulatory mechanisms are involved, including alterations to miRNA biogenesis and epigenetic controls. For example, altered epigenetic control of *SLIT2* and *SLIT3* has also been reported in lung cancer with hypermethylation of *SLIT2* and to a lesser extent *SLIT3* [56], [57]. Thus, epigenetic silencing is an alternative or additional ‘hit’, and strengthens the case for miR-218 and its host genes as potential tumour suppressor genes.

Additional support for a role for miR-218 in NSCLC comes from the potential association between miR-218 downregulation and cigarette smoke exposure. Further, Schembri *et al* has also demonstrated a link between miR-218 downregulation and smoking; exposing human bronchial epithelial cells to cigarette smoke extract decreased miR-218 expression levels [58]. Our observation that in both subtypes of NSCLC, miR-218 expression is significantly reduced in subjects with a history of cigarette smoking, provides additional support the notion that miR-218 may be involved in tobacco-related carcinogenesis [58]. However, the number of never smokers used in this study is insufficient to confirm this relationship and further investigation is required with larger cohorts and functional validation to explore a causative relationship. We also examined whether the decrease in miR-218 expression was associated with asbestos, a well known lung carcinogen, but found no significant correlation. As miR-218 may play a role in metastasis, with Leite *et al* reporting high levels of miR-218 in high grade prostate cancer compared to significantly reduced levels in metastatic prostate cancers [59], we examined the survival of our cases with miR-218 dysregulation. In lung cancer, we found no relationship between miR-218 expression levels and NSCLC recurrence or patient survival.

MiR-218 has also been shown to be downregulated in cervical carcinoma through the action of the HPV 16 E6 oncogene and may be important in early cervical tumourigenesis [25]. We have previously identified a limited association for HPV infection in lung cancer, with reports of HPV prevalence ranging from 5–22% [60], [61]. We did not find any link between HPV status and miR-218 expression but one reason may be that neither of our HPV positive samples were HPV type 16, and reduced miR-218 has only been reported with the HPV 16 – E6 oncogene. However, the small numbers preclude strong conclusions and more samples need testing. MiR-218 has also been reported to be reduced in gastric cancer, with reduced expression linked to *Helicobacter pylori* infection and carcinogenesis [62].

Target prediction found miR-218 can target numerous oncogenes, such as *KIT*, *RET*, *BCL9*, *DCUN1D1* and *PDGFRA*. Gene ontology and functional annotation clustering of the predicted targets revealed enrichment of genes involved in cell adhesion, protein modification, development, cell signalling and regulation of transcription, all processes that may contribute to carcinogenesis. These findings were reflected in the Ingenuity Pathway Analysis, with miR-218 potentially regulating genes involved in cancer-related biological functions and three recognised cancer signalling pathways. Focused pathway analysis also revealed that miR-218 may regulate genes directly and indirectly related to *MYC* and *SRC*, two well characterised oncogenes [20], [21], [63], [64], [65]. Recent studies have begun to authenticate miR-218 target genes, including *LAMB3* (laminin-5 β3) [25], *ECOP* (Epidermal growth factor receptor-coamplified and overexpressed protein) [58], [62] and transcription factor *MAGF* (v-maf musculoaponeurotic fibrosarcoma oncogene homolog G) [58].

In summary, arrayCGH cancer datasets are increasingly available with unbiased probes or selected cancer associated genes. We combined bioinformatic and experimental approaches to test whether miRNAs located in regions of gene dosage alteration had concordant altered expression, and therefore of importance. With this approach we identified putative tumour suppressor miR-218, experimentally confirmed its downregulation in NSCLC, and provided additional support for an association between reduced expression and cigarette smoke exposure. In addition, we identified relevant cancer-related target genes and pathways targeted by miR-218, supporting a potential role as a tumour suppressor gene for NSCLC, especially SCCs. Nonetheless, further evidence including structural mutations and demonstrable tumour suppressor functions should be gathered before miR-218 can be appropriately designated as a tumour-suppressor gene for lung cancer.

## Supporting Information

Figure S1 Relationship between host gene copy number, host gene expression and miR-218 expression in lung adenocarcinomas and squamous cell carcinomas. *p = 0.05. (a) No significant Pearson correlation was observed for host gene copy number and miR-218 expression in SCCs (SLIT2: −0.282, p = 0.257; SLIT3: −0.111 p = 0.661) or ACs (SLIT2: −0.078 p = 0.737; SLIT3: −0.107, p = 0.646). (b) No significant Pearson correlation was observed for host gene copy number and expression in SCCs (SLIT2: −0.389, p = 0.111; SLIT3: −0.265 p = 0.288) or ACs (SLIT2: 0.009 p = 0.970; SLIT3: 0.249, p = 0.304). (c) A significant Pearson correlation was observed between miR-218 expression and SLIT2 expression in SCCs (0.496, p = 0.036) but not ACs (0.378, p = 0.111). No significant correlation was observed between miR-218 expression and SLIT3 expression (SCC: 0.459, p = 0.055; AC: 0.297, p = 0.216).
(1.42 MB TIF)

Figure S2 MiR-218, SLIT2 and SLIT3 expression and SLIT2 and SLIT3 copy number in lung squamous cell carcinomas (a) and adenocarcinomas (b). Complete concordance (loss of both host genes plus a decrease in miR-218 expression) between SLIT2 and SLIT3 copy number and miR-218, SLIT2 and SLIT3 expression, was observed in 9/19 (47.4%) ACs and 10/18 (55.6%) SCCs. For one AC and two SCCs, miR-218 expression was increased despite reduced host gene copy numbers and expression.
(0.30 MB TIF)

Figure S3 Ingenuity Pathway Analysis miR-218. Core analysis for 578 predicted miR-218 target genes. (a) Top 20 biological functions. Threshold bar is set at significance of p<0.05 and -log(p-value) of 1.3. (b) Top 20 canonical pathways. Threshold bar is set at significance of p<0.05 and -log(p-value) of 1.3. Line illustrates the ratio of miR-218 target genes in the pathway divided by the total number of genes in the pathway. Red circles highlight cancer-associated functions or pathways.
(2.24 MB TIF)

Figure S4 Network of enriched miR-218 target genes linked to the biological functions of gene expression, cancer and cell morphology identified from Ingenuity Pathway Analysis. MiR-218 may target genes (coloured orange) directly and indirectly linked to two well known oncogenes, MYC and SRC (coloured red). For clarity, additional miRNAs and their targets involved in this network were removed therefore only 9 of the 11 miR-218 targets are shown. RELN is also a predicted target of miR-218 but was not included in the enriched gene list.
(2.29 MB TIF)

Table S1 Selected thresholds for identification of chromosomal aberrations in arrayCGH data. Abbreviations: SCC, Squamous Cell Carcinoma; AC, Adenocarcinoma; FC, Fold change; ACE, Analysis of Copy Errors; FDR, False Discovery Rate.
(0.03 MB DOC)

Table S2 ArrayCGH Publications used for miRNA prioritisation. Abbreviations: SCC, Squamous Cell Carcinoma; AC, Adenocarcinoma; AdSq, Adenosquamous Carcinoma; BAC, Bronchioalveolar Carcinoma; SCLC, Small Cell Lung Carcinoma; LC, Large Cell Carcinoma.
(0.03 MB DOC)

Table S3 MiRNA Expression studies used for miRNA prioritisation. Abbreviations: SCC, Squamous Cell Carcinoma; AC, Adenocarcinoma; AdSq, Adenosquamous Carcinoma; LC, Large Cell Carcinoma.
(0.04 MB DOC)

Table S4 Primers for host gene qRT-PCR
(0.03 MB DOC)

Table S5 Candidate miRNAs identified from positional arrayCGH analysis. Abbreviations: AC, Adenocarcinoma; SCC, Squamous Cell Carcinoma.
(0.12 MB DOC)

Table S6 Candidate miRNAs identified from public arrayCGH data.
(0.07 MB DOC)

Table S7 Summary of arrayCGH analysis for mir-218-1 and mir-218-2.
(0.07 MB DOC)

Table S8 Summary of miR-218 expression and SLIT2/SLIT3 expression and copy number changes. Abbreviations: FC, Fold Change; SCC, Squamous Cell Carcinoma; AC, Adenocarcinoma.
(0.07 MB DOC)

Table S9 Enriched miR-218 target gene groups identified by gene functional classification in DAVID.
(0.03 MB DOC)

Table S10 Top 10 networks for predicted miR-218 target genes from ingenuity pathway analysis.
(0.03 MB DOC)
