# Supplementary figures and images for: MicroRNA-218 Is Deleted and Downregulated in Lung Squamous Cell Carcinoma
Source: PLoS One. 2010 Sep 3;5(9):e12560. doi: 10.1371/journal.pone.0012560 (PMC2933228; doi:10.1371/journal.pone.0012560)

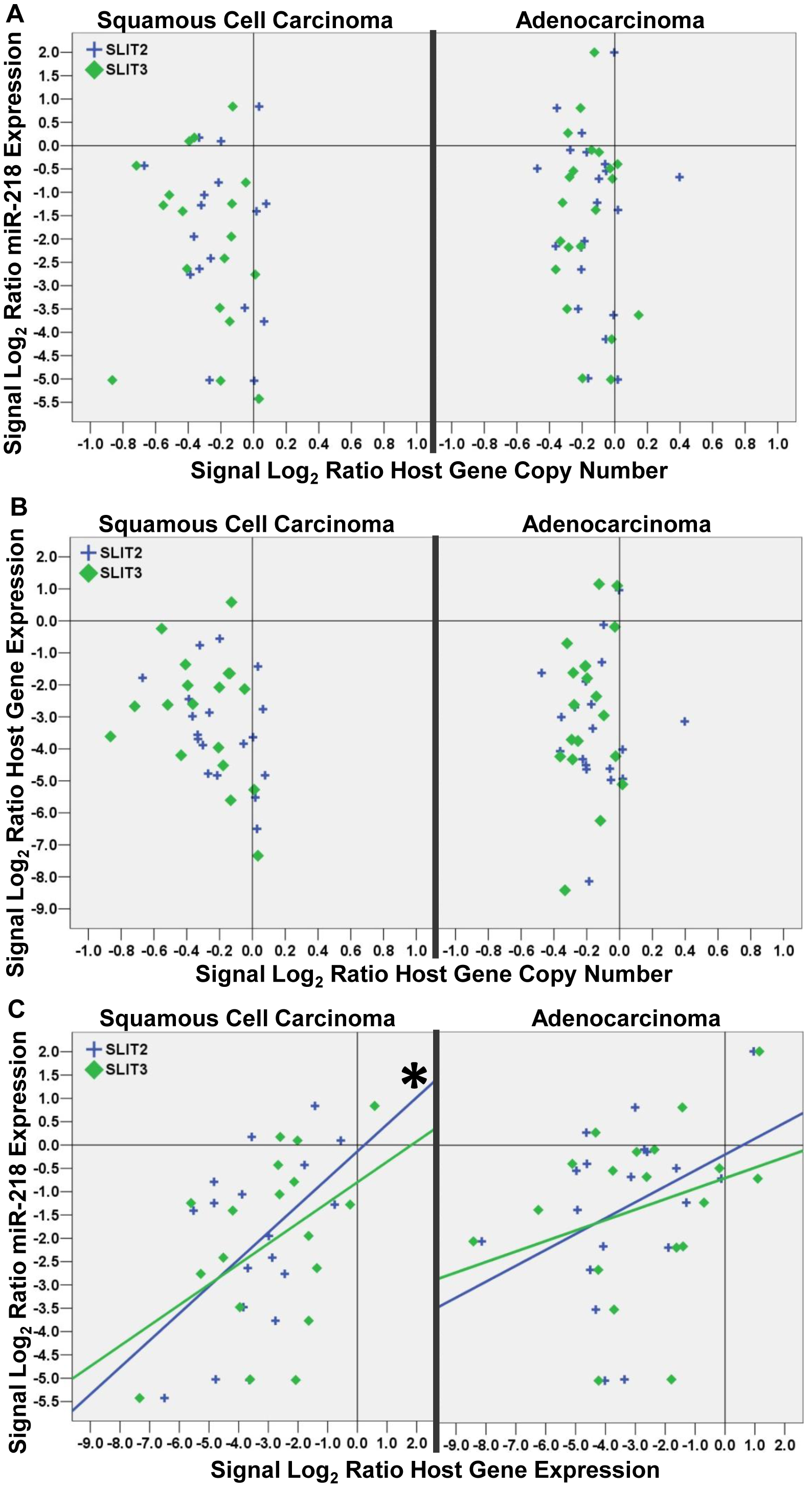

Supplement: Figure S1 — Relationship between host gene copy number, host gene expression and miR-218 expression in lung adenocarcinomas and squamous cell carcinomas. *p = 0.05. (a) No significant Pearson correlation was observed for host gene copy number and miR-218 expression in SCCs (SLIT2: −0.282, p = 0.257; SLIT3: −0.111 p = 0.661) or ACs (SLIT2: −0.078 p = 0.737; SLIT3: −0.107, p = 0.646). (b) No significant Pearson correlation was observed for host gene copy number and expression in SCCs (SLIT2: −0.389, p = 0.111; SLIT3: −0.265 p = 0.288) or ACs (SLIT2: 0.009 p = 0.970; SLIT3: 0.249, p = 0.304). (c) A significant Pearson correlation was observed between miR-218 expression and SLIT2 expression in SCCs (0.496, p = 0.036) but not ACs (0.378, p = 0.111). No significant correlation was observed between miR-218 expression and SLIT3 expression (SCC: 0.459, p = 0.055; AC: 0.297, p = 0.216). (1.42 MB TIF) [file pone.0012560.s001.tif]

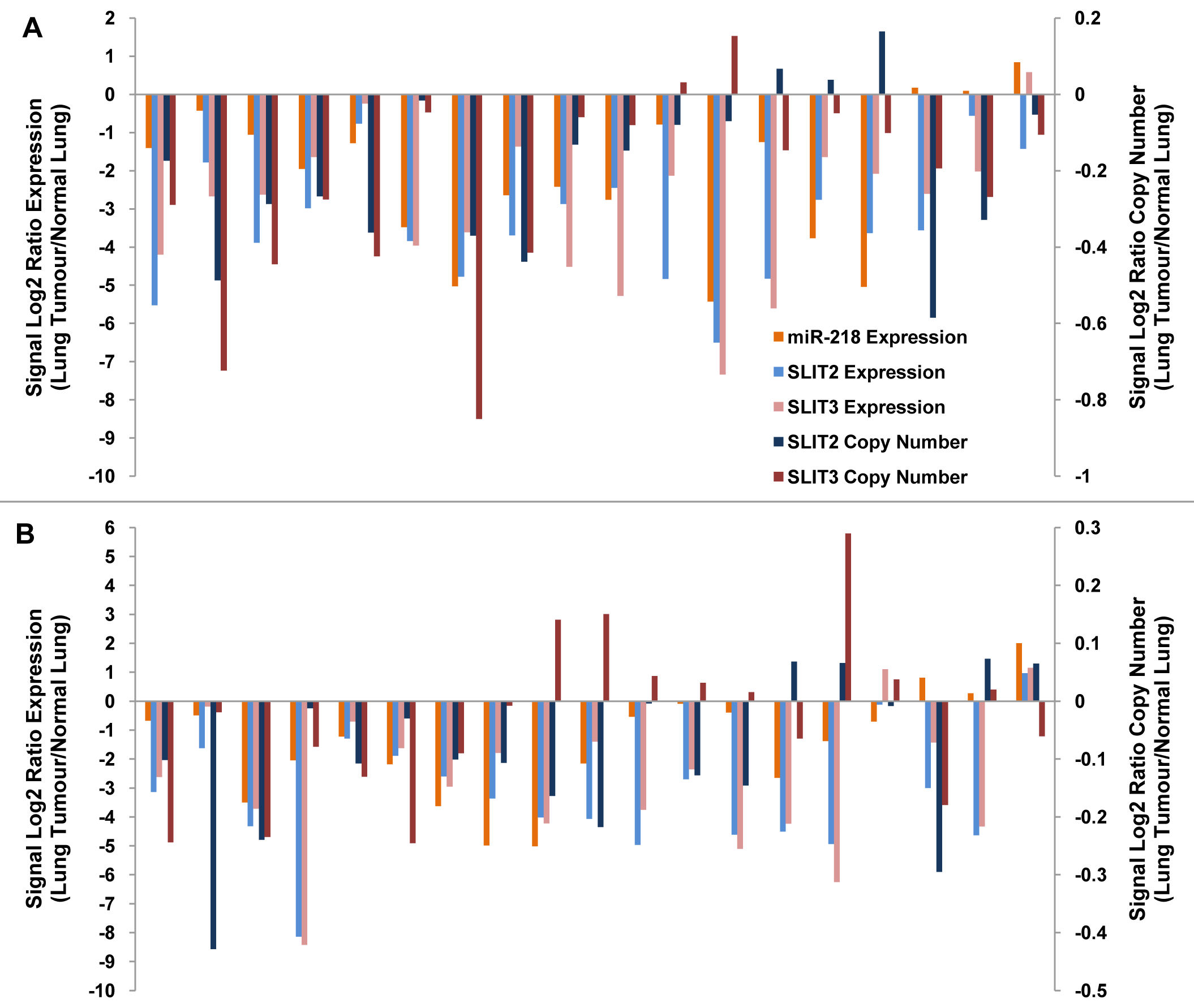

Supplement: Figure S2 — MiR-218, SLIT2 and SLIT3 expression and SLIT2 and SLIT3 copy number in lung squamous cell carcinomas (a) and adenocarcinomas (b). Complete concordance (loss of both host genes plus a decrease in miR-218 expression) between SLIT2 and SLIT3 copy number and miR-218, SLIT2 and SLIT3 expression, was observed in 9/19 (47.4%) ACs and 10/18 (55.6%) SCCs. For one AC and two SCCs, miR-218 expression was increased despite reduced host gene copy numbers and expression. (0.30 MB TIF) [file pone.0012560.s002.tif]

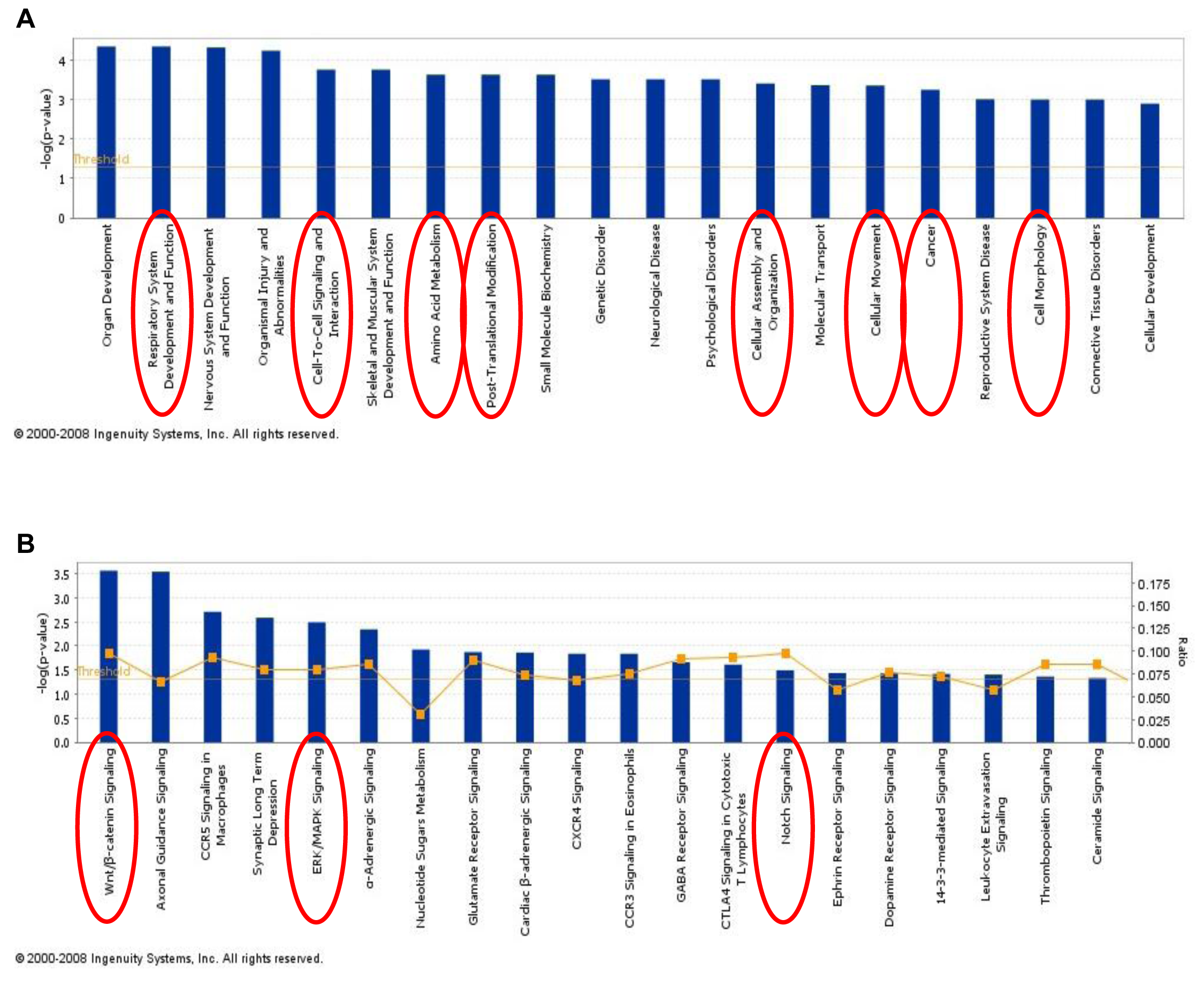

Supplement: Figure S3 — Ingenuity Pathway Analysis miR-218. Core analysis for 578 predicted miR-218 target genes. (a) Top 20 biological functions. Threshold bar is set at significance of p<0.05 and -log(p-value) of 1.3. (b) Top 20 canonical pathways. Threshold bar is set at significance of p<0.05 and -log(p-value) of 1.3. Line illustrates the ratio of miR-218 target genes in the pathway divided by the total number of genes in the pathway. Red circles highlight cancer-associated functions or pathways. (2.24 MB TIF) [file pone.0012560.s003.tif]

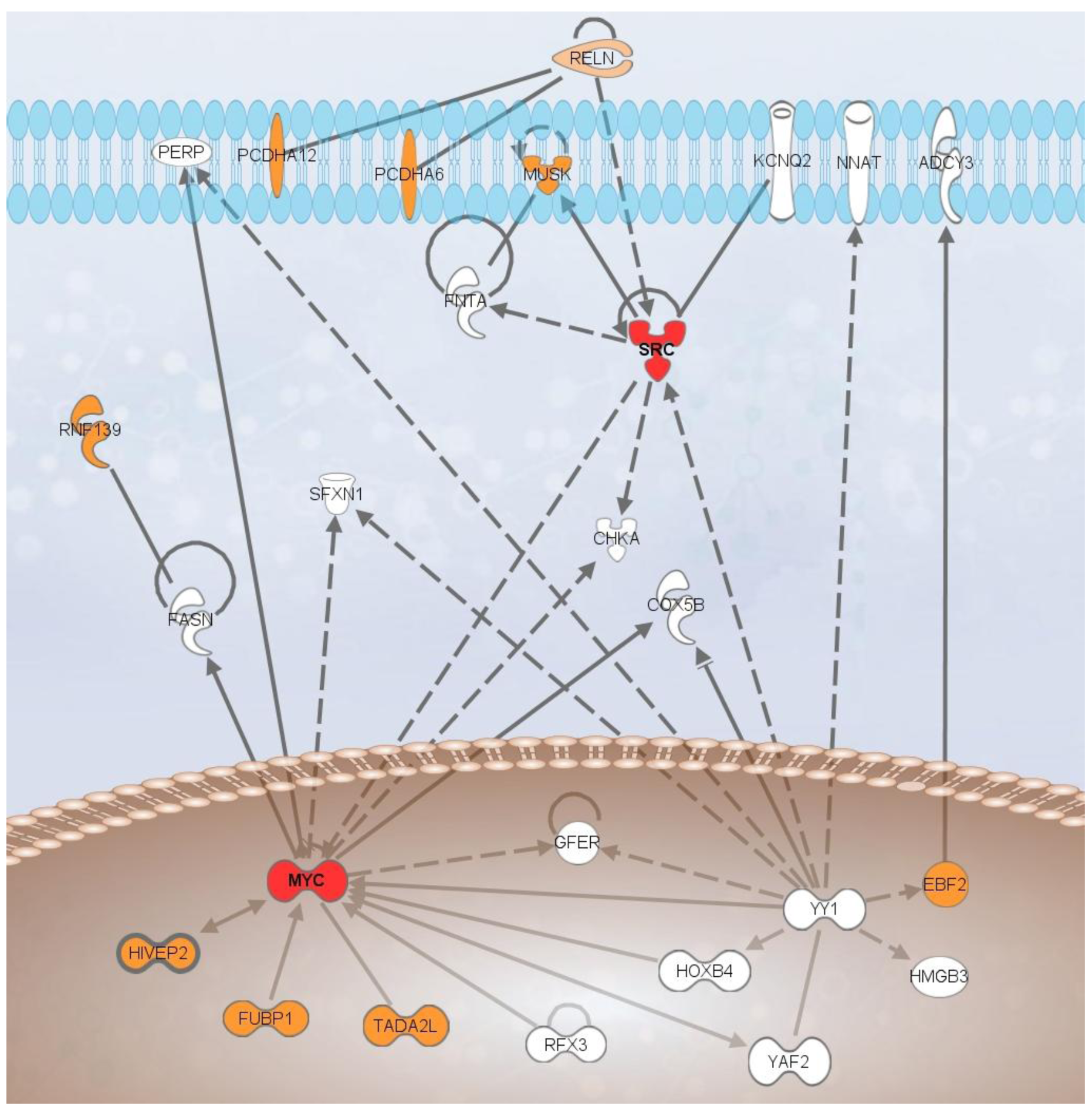

Supplement: Figure S4 — Network of enriched miR-218 target genes linked to the biological functions of gene expression, cancer and cell morphology identified from Ingenuity Pathway Analysis. MiR-218 may target genes (coloured orange) directly and indirectly linked to two well known oncogenes, MYC and SRC (coloured red). For clarity, additional miRNAs and their targets involved in this network were removed therefore only 9 of the 11 miR-218 targets are shown. RELN is also a predicted target of miR-218 but was not included in the enriched gene list. (2.29 MB TIF) [file pone.0012560.s004.tif]
