# Supplementary material for: MicroRNA-218 Is Deleted and Downregulated in Lung Squamous Cell Carcinoma
Source: PLoS One. 2010 Sep 3;5(9):e12560. doi: 10.1371/journal.pone.0012560 (PMC2933228; doi:10.1371/journal.pone.0012560)
Supplement: Table S1 — Selected thresholds for identification of chromosomal aberrations in arrayCGH data. Abbreviations: SCC, Squamous Cell Carcinoma; AC, Adenocarcinoma; FC, Fold change; ACE, Analysis of Copy Errors; FDR, False Discovery Rate. (0.03 MB DOC) [file pone.0012560.s005.doc]

| **Histotype** | ***Autosomal Chromosomes*** | | | ***X Chromosome*** | | |
| --- | --- | --- | --- | --- | --- | --- |
|  | **FC** | **CGH Explorer - ACE** | | **FC** | **CGH Explorer - ACE** | |
|  |  | **% Genes Altered** | **FDR** |  | **% Genes Altered** | **FDR** |
| SCC | ± 1.2 | 46.4 | 0.0009 | ± 1.2 | 44.2 | 0.0009 |
| AC | ± 1.2 | 30.0 | 0.0008 | ± 1.2 | 31.3 | 0.0007 |
