# Supplementary material for: MicroRNA-218 Is Deleted and Downregulated in Lung Squamous Cell Carcinoma
Source: PLoS One. 2010 Sep 3;5(9):e12560. doi: 10.1371/journal.pone.0012560 (PMC2933228; doi:10.1371/journal.pone.0012560)
Supplement: Table S2 — ArrayCGH Publications used for miRNA prioritisation. Abbreviations: SCC, Squamous Cell Carcinoma; AC, Adenocarcinoma; AdSq, Adenosquamous Carcinoma; BAC, Bronchioalveolar Carcinoma; SCLC, Small Cell Lung Carcinoma; LC, Large Cell Carcinoma. (0.03 MB DOC) [file pone.0012560.s006.doc]

| **First Author** | **Year** | **Cohort** | **Platform** |
| --- | --- | --- | --- |
| **Garnis [33]** | 2005 | 1 primary SCC | SMRT (submegabase resolution tiling set) array with 32,433 overlapping BAC clones |
| **Tonon [34]** | 2005 | Primary tumours (18 AC; 26 SCC) and cell lines (24 AC; 2 SCC; 3AdSq; 1 BAC; 4 other) | cDNA (14k, Human clone 1 set, Agilent) and oligonucleotide (22k, Human 1A V2, Agilent) arrays |
| **Zhao [35]** | 2005 | Primary tumours (37 AC; 10 SCC; 1 AC/BAC; 1 BAC; 19 SCLC) and cell lines (12 AC, 3 SCC; 1 AdSq; 1 BAC; 3 LC; 5 SCLC) | CentXba and CentHind SNP arrays (115,000 SNPs, Affymetrix) |
