# Supplementary material for: MicroRNA-218 Is Deleted and Downregulated in Lung Squamous Cell Carcinoma
Source: PLoS One. 2010 Sep 3;5(9):e12560. doi: 10.1371/journal.pone.0012560 (PMC2933228; doi:10.1371/journal.pone.0012560)
Supplement: Table S3 — MiRNA Expression studies used for miRNA prioritisation. Abbreviations: SCC, Squamous Cell Carcinoma; AC, Adenocarcinoma; AdSq, Adenosquamous Carcinoma; LC, Large Cell Carcinoma. (0.04 MB DOC) [file pone.0012560.s007.doc]

| **First Author** | **Year** | **Cohort** | **Method for miRNA profiling** |
| --- | --- | --- | --- |
| **Johnson [7]** | 2005 | 12 lung cancers | mirVana miRNA array (Ambion) and Northern blot |
| **Takamizawa [6]** | 2004 | 143 NSCLCs (105 AC; 25 SCC; 9 LC; 4 AdSq) | Northern blot and precursor qRT-PCR |
| **Volinia [36]** | 2006 | 123 paired primary lung cancers | miRNA microarray (102) with 352 probes |
| **Yanaihara [8]** | 2006 | 104 paired primary NSCLCs (65 AC; 39 SCC) | miRNA microarray (102) with 352 probes |
