# Supplementary material for: MicroRNA-218 Is Deleted and Downregulated in Lung Squamous Cell Carcinoma
Source: PLoS One. 2010 Sep 3;5(9):e12560. doi: 10.1371/journal.pone.0012560 (PMC2933228; doi:10.1371/journal.pone.0012560)
Supplement: Table S4 — Primers for host gene qRT-PCR (0.03 MB DOC) [file pone.0012560.s008.doc]

| **Primer** | **Sequence** |
| --- | --- |
| *SLIT2* Forward | TCCACTCTATGTAGGAGGCATGC |
| *SLIT2* Reverse | CGCAGAGATGCCACGTTACTC |
| *SLIT3* Forward | GTGCCACATCTCAGACCAAGG |
| *SLIT3* Reverse | GCGGATCACCTCTCGGACTA |
| 18s rRNA Forward | CGGCTACCACATCCAAGGAA |
| 18s rRNA Reverse | GCTGGAATTACCGCGGCT |
| *BAT1* Forward | CAAGATCCAATGGAGATCTTCGT |
| *BAT1* Reverse | TCGTTGTCCTTCAGTTTCACGTAG |
| *ACTN4* Forward | AGCGCAAGACCTTCACGG |
| *ACTN4* Reverse | TCATCAATGTTCTCGATCTGTGTG |
