# Supplementary material for: MicroRNA-218 Is Deleted and Downregulated in Lung Squamous Cell Carcinoma
Source: PLoS One. 2010 Sep 3;5(9):e12560. doi: 10.1371/journal.pone.0012560 (PMC2933228; doi:10.1371/journal.pone.0012560)
Supplement: Table S5 — Candidate miRNAs identified from positional arrayCGH analysis. Abbreviations: AC, Adenocarcinoma; SCC, Squamous Cell Carcinoma. (0.12 MB DOC) [file pone.0012560.s009.doc]

| **Precursor miRNA** | **miRNA Locus** | **Host Gene** | **Location** | **AC** | **SCC** |
| --- | --- | --- | --- | --- | --- |
| **mir-34a** | 1p36.22 |  | Intergenic |  | Loss |
| **mir-554** | 1q21.3 | *TUFT1* | Intron |  | Gain |
| **mir-92b** | 1q22 |  | Intergenic | Gain | Gain |
| **mir-765** | 1q23.1 | *ARHGEF11* | Intron | Gain | Gain |
| **mir-557** | 1q24.2 |  | Intergenic | Gain |  |
| **mir-214** | 1q24.3 | *DNM3* | Antisense | Gain |  |
| **mir-199a-2** |  |  |  | Gain |  |
| **mir-181b-1** | 1q31.3 |  | Intergenic | Gain | Gain |
| **mir-181a-1** | 1q31.3 |  | Intergenic | Gain | Gain |
| **mir-217** | 2p16.1 |  | Intergenic |  | Gain |
| **mir-216** | 2p16.1 |  | Intergenic |  | Gain |
| **mir-563** | 3p25.1 |  | Intergenic |  | Loss |
| **mir-26a-1** | 3p22.3 | *CTDSPL* | Intron |  | Loss |
| **mir-565** | 3p21.31 |  | Intergenic |  | Loss |
| **mir-566** | 3p21.31 | *SEMA3F* | Intron |  | Loss |
| **let-7g** | 3p21.2 | *WDR82* | Intron |  | Loss |
| **mir-198** | 3q13.33 | *FSTL1* | Exon 11 |  | Gain |
| **mir-15b** | 3q25.33 | *SMC4* | Intron |  | Gain |
| **mir-16-2** |  |  |  |  | Gain |
| **mir-551b** | 3q26.2 |  | Intergenic |  | Gain |
| **mir-569** | 3q26.2 | *TNIK* | Intron |  | Gain |
| **mir-571** | 4p16.3 | *ZNF141* | Intron |  | Loss |
| **mir-95** | 4p16.1 | *ABLIM2* | Intron |  | Loss |
| **mir-572** | 4p16.1 |  | Intergenic |  | Loss |
| **mir-218-1** | 4p15.31 | *SLIT2* | Intron |  | Loss |
| **mir-578** | 4q32.3 | *CPE* | Intron |  | Loss |
| **mir-579** | 5p13.3 | *ZFR* | Intron | Gain | Gain |
| **mir-580** | 5p13.2 | *LMBRD2* | Intron | Gain | Gain |
| **mir-581** | 5q11.2 | *ARL15* | Intron |  | Loss |
| **mir-583** | 5q15 |  | Intergenic |  | Loss |
| **mir-103-1** | 5q34 | *PANK3* | Intron |  | Loss |
| **mir-218-2** | 5q35.1 | *SLIT3* | Intron |  | Loss |
| **mir-340** | 5q35.3 | *RNF130* | Intron |  | Loss |
| **mir-589** | 7p22.1 | *FBXL18* | Intron | Gain | Gain |
| **mir-550-1** | 7p15.1 | *ZNRF2* | Intron | Gain |  |
| **mir-591** | 7q21.3 | *SLC25A13* | Intron |  | Gain |
| **mir-25** | 7q22.1 | *MCM7* | Intron |  | Gain |
| **mir-93** |  |  |  |  | Gain |
| **mir-106b** |  |  |  |  | Gain |
| **mir-335** | 7q32.2 | *MEST* | Intron | Gain | Gain |
| **mir-596** | 8p23.3 |  | Intergenic |  | Loss |
| **mir-597** | 8p23.1 | *TNKS* | Intron |  | Loss |
| **mir-383** | 8p22 |  | Intergenic |  | Loss |
| **mir-599** | 8q22.2 |  | Intergenic |  | Gain |
| **mir-548a-3** | 8q22.3 |  | Intergenic | Gain | Gain |
| **mir-151** | 8q24.3 | *PTK2* | Intron | Gain | Gain |
| **mir-661** | 8q24.3 | *PLEC1* | Intron |  | Gain |
| **mir-31** | 9p21.3 |  | Intergenic |  | Loss |
| **mir-605** | 10q21.1 | *PRKG1* | Intron |  | Loss |
| **mir-107** | 10q23.31 | *PANK1* | Intron |  | Loss |
| **mir-202** | 10q26.3 | *Putative transcript* | Intron |  | Loss |
| **mir-200c** | 12p13.31 |  | Intergenic |  | Gain |
| **mir-141** | 12p13.31 |  | Intergenic |  | Gain |
| **mir-613** | 12p13.2 | *APOLD1* | Intron |  | Gain |
| **mir-614** | 12p13.1 |  | Intergenic |  | Gain |
| **mir-615** | 12q13.13 | *HOXC5* | Intron |  | Gain |
| **mir-616** | 12q13.3 | *DDIT3* | Intron |  | Gain |
| **mir-622** | 13q31.3 |  | Intergenic |  | Loss |
| **mir-17** | 13q31.3 | *Q75NE7 / MIRHG1* | Intron / Exon 2 |  | Loss |
| **mir-17** |  |  |  |  | Loss |
| **mir-18a** |  |  |  |  | Loss |
| **mir-18a** |  |  |  |  | Loss |
| **mir-19a** |  |  |  |  | Loss |
| **mir-20a** |  |  |  |  | Loss |
| **mir-19b-1** |  |  |  |  | Loss |
| **mir-92-1** |  |  |  |  | Loss |
| **mir-152** | 17q21.32 | *COPZ2* | Intron |  | Gain |
| **mir-10a** | 17q21.32 |  | Intergenic |  | Gain |
| **mir-21** | 17q23.2 |  | Intergenic | Gain | Gain |
| **mir-634** | 17q24.2 | *PRKCA* | Intron | Gain | Gain |
| **mir-548d-2** | 17q24.2 | *PITPNC1* | Antisense |  | Gain |
| **mir-635** | 17q24.2 | *WIPI1* | Intron | Gain |  |
| **mir-636** | 17q25.2 | *SFRS2* | Intron | Gain | Gain |
| **mir-639** | 19p13.12 | *GPSN2* | Exon 1 |  | Gain |
| **mir-1-1** | 20q13.33 | *C20orf166* | Intron | Gain |  |
| **mir-133a-2** | 20q13.33 |  | Intergenic | Gain |  |
| **mir-647** | 20q13.33 | *UCKL1* | Intron | Gain |  |
| **mir-648** | 22q11.21 |  | Intergenic |  | Gain |
| **mir-130b** | 22q11.21 |  | Intergenic |  | Gain |
| **mir-650** | 22q11.22 | *IGLC3* | Intron |  | Gain |
| **mir-362** | 23p11.23 | *CLCN5* | Intron | Loss |  |
| **mir-501** |  |  |  | Loss |  |
| **mir-660** |  |  |  | Loss |  |
| **mir-502** |  |  |  | Loss |  |
| **mir-98** | 23p11.22 | *HUWE1* | Intron |  | Loss |
| **let-7f-2** |  |  |  |  | Loss |
| **mir-384** | 23q21.1 |  | Intergenic | Loss | Loss |
| **mir-325** | 23q21.1 |  | Intergenic |  | Loss |
