# Supplementary material for: MicroRNA-218 Is Deleted and Downregulated in Lung Squamous Cell Carcinoma
Source: PLoS One. 2010 Sep 3;5(9):e12560. doi: 10.1371/journal.pone.0012560 (PMC2933228; doi:10.1371/journal.pone.0012560)
Supplement: Table S6 — Candidate miRNAs identified from public arrayCGH data. (0.07 MB DOC) [file pone.0012560.s010.doc]

| **Precursor miRNA** | **miRNA Locus** | **Host Gene** | **Location** | **Genomic Change** |
| --- | --- | --- | --- | --- |
| **mir-30e** | 1p34.2 | *NFYC* | Intron | Gain |
| **mir-30e** |  |  |  | Gain |
| **mir-30c-1** |  |  |  | Gain |
| **mir-101-1** | 1p31.3 |  | Intergenic | Gain |
| **mir-181b-1** | 1p31.3 |  | Intergenic | Gain |
| **mir-181a-1** | 1p31.3 |  | Intergenic | Gain |
| **mir-558** | 2p22.3 | *BIRC6* | Intron | Loss |
| **mir-217** | 2p16.1 |  | Intergenic | Gain |
| **mir-216** | 2p16.1 |  | Intergenic | Gain |
| **mir-566** | 3p21.31 | *SEMA3F* | Intron | Loss |
| **mir-551b** | 3q26.2 |  | Intergenic | Gain |
| **mir-579** | 5p13.3 | *ZFR* | Intron | Gain |
| **mir-580** | 5p13.2 | *LMBRD2* | Intron | Gain |
| **mir-581** | 5q11.2 | *ARL15* | Intron | Loss |
| **mir-449** | 5q11.2 | *CDC20B* | Intron | Loss |
| **mir-449b** |  |  |  | Loss |
| **mir-582** | 5q12.1 | *PDE4D* | Intron | Loss |
| **mir-9-2** | 5q14.3 |  | Intergenic | Loss |
| **mir-583** | 5q15 |  | Intergenic | Loss |
| **mir-584** | 5q32 | *SH3TC2* | Intron | Loss |
| **mir-143** | 5q32 |  | Intergenic | Loss |
| **mir-145** | 5q32 |  | Intergenic | Loss |
| **mir-378** | 5q32 | *PPARGC1B* | Intron | Loss |
| **mir-146a** | 5q33.3 |  | Intergenic | Loss |
| **mir-103-1** | 5q34 | *PANK3* | Intron | Loss |
| **mir-218-2** | 5q34 | *SLIT3* | Intron | Loss |
| **mir-585** | 5q35.1 |  |  | Loss |
| **mir-340** | 5q35.3 | *RNF130* | Intron | Loss |
| **mir-548a-1** | 6p22.3 |  | Intergenic | Gain |
| **mir-206** | 6p12.2 |  | Intergenic | Gain |
| **mir-133b** | 6p12.2 |  | Intergenic | Gain |
| **mir-548b** | 6q22.31 | *FAM184A* | Intron | Loss |
| **mir-588** | 6q22.32 |  | Intergenic | Loss |
| **mir-486** | 8p11.21 | *ANK1* | Intron | Gain |
| **mir-124a-2** | 8q12.3 |  | Intergenic | Gain |
| **mir-548d-1** | 8q24.13 | *ATAD2* | Intron | Gain |
| **mir-31** | 9p21.3 |  | Intergenic | Loss |
| **mir-107** | 10q23.31 | *PANK1* | Intron | Loss |
| **mir-613** | 12p13.2 | *APOLD1* | Intron | Gain |
| **mir-614** | 12p13.1 |  | Intergenic | Gain |
| **mir-26a-2** | 12q14.1 | *CTDSP2* | Intron | Gain |
| **mir-16-1** | 13q14.2 | *DLEU2* | Intron | Loss |
| **mir-15a** |  |  |  | Loss |
| **mir-33b** | 17p11.2 | *SREBF1* | Intron | Gain |
| **mir-296** | 20q13.32 |  | Intergenic | Gain |
| **mir-646** | 20q13.33 | *RP5-1043L13.1* | Intron | Gain |
| **mir-99a** | 21q21.1 |  | Intergenic | Gain |
| **let-7c** | 21q21.1 |  | Intergenic | Gain |
| **mir-125b-2** | 21q21.1 |  | Intergenic | Gain |
| **mir-649** | 22q11.21 |  | Intergenic | Gain |
| **mir-130b** | 22q11.21 |  | Intergenic | Gain |
