# Supplementary material for: MicroRNA-218 Is Deleted and Downregulated in Lung Squamous Cell Carcinoma
Source: PLoS One. 2010 Sep 3;5(9):e12560. doi: 10.1371/journal.pone.0012560 (PMC2933228; doi:10.1371/journal.pone.0012560)
Supplement: Table S7 — Summary of arrayCGH analysis for mir-218-1 and mir-218-2. (0.07 MB DOC) [file pone.0012560.s011.doc]

|  | **Gene Name** | **Probe Position** | | | **Fold Change** | | | | | **ACE** | |
| --- | --- | --- | --- | --- | --- | --- | --- | --- | --- | --- | --- |
|  |  |  |  |  | **% Tumours**  **(FC ≥ ±1.2)** | | **Min** | **Max** | **Average** | **% Tumours** | |
|  |  | **Chr** | **Start** | **End** | **AMP** | **DEL** |  |  |  | **AMP** | **DEL** |
| **SCCs** | | | | | | | | | | | |
|  | *SLIT2* | 4 | 19933977 | 19934036 | 1.7 | 51.7 | -1.91 | 1.33 | -1.25 | 3.3 | 60.0 |
|  | *SLIT2* | 4 | 20003994 | 20004053 | 1.7 | 36.7 | -1.95 | 1.21 | -1.15 | 3.3 | 60.0 |
|  | *mir-218-1* | 4 | 20138996 | 20139105 |  |  |  |  |  |  |  |
|  | *SLIT2* | 4 | 20150226 | 20150285 | 1.7 | 30.0 | -1.45 | 1.29 | -1.11 | 3.3 | 58.3 |
|  | *SLIT2* | 4 | 20229748 | 20229805 | 3.3 | 28.3 | -1.66 | 1.33 | -1.10 | 3.3 | 58.3 |
|  |  |  |  |  |  |  |  |  |  |  |  |
|  | *SLIT3* | 5 | 168021415 | 168021474 | 1.7 | 30.0 | -1.62 | -1.14 | -1.14 | 1.7 | 53.3 |
|  | *SLIT3* | 5 | 168066035 | 168066094 | 0.0 | 28.3 | -1.56 | -1.13 | -1.13 | 1.7 | 53.3 |
|  | *SLIT3* | 5 | 168097591 | 168097650 | 26.7 | 36.7 | -1.89 | -1.04 | -1.04 | 1.7 | 53.3 |
|  | *SLIT3* | 5 | 168112876 | 168112935 | 3.3 | 70.0 | -2.23 | -1.30 | -1.30 | 1.7 | 53.3 |
|  | *mir-218-2* | 5 | 168127729 | 168127838 |  |  |  |  |  |  |  |
|  | *SLIT3* | 5 | 168209957 | 168210016 | 0.0 | 25.0 | -1.61 | -1.11 | -1.11 | 1.7 | 53.3 |
|  | *SLIT3* | 5 | 168371725 | 168371784 | 6.7 | 50.0 | -2.17 | -1.21 | -1.21 | 1.7 | 53.3 |
|  | *SLIT3* | 5 | 168402833 | 168402892 | 0.0 | 76.7 | -2.46 | -1.48 | -1.48 | 1.7 | 53.3 |
|  | *SLIT3* | 5 | 168487342 | 168487401 | 3.3 | 15.0 | -1.53 | -1.02 | -1.02 | 1.7 | 53.3 |
| **Adenocarcinomas** | | | | | | | | | | | |
|  | *SLIT2* | 4 | 19933977 | 19934036 | 12.5 | 8.3 | -1.94 | 1.41 | 1.00 | 11.1 | 11.1 |
|  | *SLIT2* | 4 | 20003994 | 20004053 | 6.9 | 22.2 | -1.51 | 2.17 | -1.06 | 11.1 | 11.1 |
|  | *mir-218-1* | 4 | 20138996 | 20139105 |  |  |  |  |  |  |  |
|  | *SLIT2* | 4 | 20150226 | 20150285 | 2.8 | 11.1 | -1.32 | 1.33 | -1.07 | 11.1 | 11.1 |
|  | *SLIT2* | 4 | 20229748 | 20229805 | 19.4 | 15.3 | -1.37 | 1.65 | 1.03 | 11.1 | 11.1 |
|  |  |  |  |  |  |  |  |  |  |  |  |
|  | *SLIT3* | 5 | 168021415 | 168021474 | 1.4 | 6.9 | -1.47 | 1.21 | -1.05 | 4.2 | 15.3 |
|  | *SLIT3* | 5 | 168066035 | 168066094 | 1.4 | 15.3 | -1.36 | 1.26 | -1.09 | 4.2 | 15.3 |
|  | *SLIT3* | 5 | 168097591 | 168097650 | 29.2 | 19.4 | -1.58 | 2.01 | 1.05 | 4.2 | 15.3 |
|  | *SLIT3* | 5 | 168112876 | 168112935 | 1.4 | 33.3 | -1.68 | 1.21 | -1.15 | 4.2 | 15.3 |
|  | *mir-218-2* | 5 | 168127729 | 168127838 |  |  |  |  |  |  |  |
|  | *SLIT3* | 5 | 168209957 | 168210016 | 2.8 | 16.7 | -1.85 | 1.39 | -1.08 | 5.6 | 15.3 |
|  | *SLIT3* | 5 | 168371725 | 168371784 | 9.7 | 9.7 | -2.08 | 1.45 | 1.01 | 5.6 | 15.3 |
|  | *SLIT3* | 5 | 168402833 | 168402892 | 11.1 | 18.1 | -1.80 | 1.54 | -1.01 | 5.6 | 15.3 |
|  | *SLIT3* | 5 | 168487342 | 168487401 | 38.9 | 2.8 | -1.47 | 1.75 | 1.15 | 5.6 | 15.3 |
