# Supplementary material for: MicroRNA-218 Is Deleted and Downregulated in Lung Squamous Cell Carcinoma
Source: PLoS One. 2010 Sep 3;5(9):e12560. doi: 10.1371/journal.pone.0012560 (PMC2933228; doi:10.1371/journal.pone.0012560)
Supplement: Table S8 — Summary of miR-218 expression and SLIT2/SLIT3 expression and copy number changes. Abbreviations: FC, Fold Change; SCC, Squamous Cell Carcinoma; AC, Adenocarcinoma. (0.07 MB DOC) [file pone.0012560.s012.doc]

| **Histology** | **FC miR-218** | **FC *SLIT2*** | | **FC *SLIT3*** | |
| --- | --- | --- | --- | --- | --- |
|  |  | **Expression** | **CN** | **Expression** | **CN** |
| SCC | -2.65 | -45.89 | -1.13 | -18.38 | -1.22 |
| SCC | -1.35 | -3.43 | -1.40 | -6.36 | -1.65 |
| SCC | -2.08 | -14.76 | -1.22 | -6.16 | -1.36 |
| SCC | -3.86 | -7.91 | -1.20 | -3.12 | -1.21 |
| SCC | -2.42 | -1.70 | -1.29 | -1.18 | -1.34 |
| SCC | -11.13 | -14.32 | -1.01 | -15.56 | -1.03 |
| SCC | -32.56 | -27.35 | -1.29 | -12.24 | -1.80 |
| SCC | -6.23 | -12.94 | -1.35 | -2.57 | -1.33 |
| SCC | -5.33 | -7.29 | -1.10 | -22.89 | -1.04 |
| SCC | -6.78 | -5.45 | -1.11 | -38.76 | -1.06 |
| SCC | -1.73 | -28.44 | -1.06 | -4.38 | 1.02 |
| SCC | -42.98 | -90.51 | -1.05 | -162.02 | 1.11 |
| SCC | -2.37 | -28.38 | 1.05 | -48.73 | -1.11 |
| SCC | -13.62 | -6.77 | 1.03 | -3.12 | -1.03 |
| SCC | -32.86 | -12.44 | 1.12 | -4.22 | -1.07 |
| SCC | 1.13 | -11.79 | -1.50 | -6.06 | -1.14 |
| SCC | 1.07 | -1.47 | -1.26 | -4.05 | -1.20 |
| SCC | 1.79 | -2.69 | -1.04 | 1.50 | -1.08 |
| AC | -1.60 | -8.84 | -1.07 | -6.16 | -1.18 |
| AC | -1.40 | -3.09 | -1.35 | -1.14 | -1.01 |
| AC | -11.31 | -20.02 | -1.18 | -13.12 | -1.18 |
| AC | -4.13 | -282.09 | -1.01 | -342.51 | -1.06 |
| AC | -2.33 | -2.45 | -1.08 | -1.63 | -1.09 |
| AC | -4.53 | -3.71 | -1.02 | -3.08 | -1.19 |
| AC | -1.10 | -6.09 | -1.07 | -7.76 | -1.06 |
| AC | -31.72 | -10.29 | -1.08 | -3.47 | -1.01 |
| AC | -32.30 | -16.22 | -1.12 | -18.77 | 1.10 |
| AC | -4.45 | -16.83 | -1.16 | -2.65 | 1.11 |
| AC | -1.46 | -31.41 | -1.00 | -13.49 | 1.03 |
| AC | -1.07 | -6.50 | -1.09 | -5.13 | 1.02 |
| AC | -1.32 | -24.59 | -1.11 | -34.54 | 1.01 |
| AC | -6.29 | -22.78 | 1.05 | -18.90 | -1.05 |
| AC | -2.60 | -30.63 | 1.05 | -75.93 | 1.22 |
| AC | -1.64 | -1.09 | -1.01 | 2.14 | 1.03 |
| AC | 1.75 | -8.04 | -1.23 | -2.69 | -1.13 |
| AC | 1.21 | -24.93 | 1.05 | -20.11 | 1.01 |
| AC | 4.00 | 1.95 | 1.05 | 2.22 | -1.04 |
