# Supplementary material for: MicroRNA-218 Is Deleted and Downregulated in Lung Squamous Cell Carcinoma
Source: PLoS One. 2010 Sep 3;5(9):e12560. doi: 10.1371/journal.pone.0012560 (PMC2933228; doi:10.1371/journal.pone.0012560)
Supplement: Table S9 — Enriched miR-218 target gene groups identified by gene functional classification in DAVID. (0.03 MB DOC) [file pone.0012560.s013.doc]

| **Gene Functional Classification Group** | **Enrichment Score** | **No. Target Genes** |
| --- | --- | --- |
| Cell adhesion, cell membrane | 4.66 | 14 |
| Ubiquitin cycle, protein modification, metabolic process | 3.67 | 9 |
| Ubiquitin cycle, protein modification | 3.30 | 5 |
| Protein kinase activity, phosphorylation, kinase activity, protein modification, signal transduction | 2.88 | 22 |
| Regulation of transcription, proto-oncogene | 2.22 | 60 |
| Ion transport | 2.04 | 5 |
| Intracellular signalling, GTP binding, Ras, GTPase activity, protein localisation, cell secretion | 2.00 | 6 |
| **Total Number of Enriched Genes** | **≥2.00** | **121** |
