# Supplementary material for: MicroRNA-218 Is Deleted and Downregulated in Lung Squamous Cell Carcinoma
Source: PLoS One. 2010 Sep 3;5(9):e12560. doi: 10.1371/journal.pone.0012560 (PMC2933228; doi:10.1371/journal.pone.0012560)
Supplement: Table S10 — Top 10 networks for predicted miR-218 target genes from ingenuity pathway analysis. (0.03 MB DOC) [file pone.0012560.s014.doc]

| ***All 578 Predicted miR-218 Targets*** | | ***121 Enriched miR-218 Predicted Targets*** | |
| --- | --- | --- | --- |
| **Network Functions** | **No. Genes** | **Network Functions** | **No. Genes** |
| Amino Acid Metabolism, Post-Translational Modification, Small Molecule Biochemistry | 30 | Amino Acid Metabolism, Post-Translational Modification, Small Molecule Biochemistry | 21 |
| Molecular Transport, Small Molecule Biochemistry, Cellular Assembly and Organization | 30 | Connective Tissue Development and Function, Skeletal and Muscular System Development and Function, Tissue Development | 16 |
| Gene Expression, Small Molecule Biochemistry, Cellular Growth and Proliferation | 28 | Amino Acid Metabolism, Post-Translational Modification, Small Molecule Biochemistry | 14 |
| Cellular Development, Respiratory System Development and Function, Skeletal and Muscular System Development and Function | 26 | Cell Cycle, Endocrine System Development and Function, Skeletal and Muscular System Development and Function | 14 |
| Behaviour, Cell-To-Cell Signaling and Interaction, Post-Translational Modification | 25 | Gene Expression, Cellular Compromise, Cellular Development | 13 |
| Cellular Movement, Cell Morphology, Digestive System Development and Function | 24 | ***Gene Expression, Cancer, Cell Morphology*** | 11 |
| Embryonic Development, Tissue Development, Cellular Development | 24 | Molecular Transport, Protein Trafficking, Cellular Assembly and Organization | 11 |
| Cell Signaling, DNA Replication, Recombination, and Repair, Nucleic Acid Metabolism | 20 | RNA Post-Transcriptional Modification, Cell-To-Cell Signaling and Interaction, Cellular Assembly and Organization | 5 |
| Cellular Development, Cell Morphology, Cellular Assembly and Organization | 20 | Gene Expression | 1 |
| RNA Damage and Repair, Amino Acid Metabolism, Drug Metabolism | 18 | Carbohydrate Metabolism, Cellular Compromise, Cellular Movement | 1 |
